# Factors associated with postpartum depression among women in Vientiane Capital, Lao People’s Democratic Republic: A cross-sectional study

**DOI:** 10.1371/journal.pone.0243463

**Published:** 2020-12-04

**Authors:** Souphalak Inthaphatha, Eiko Yamamoto, Viengsakhone Louangpradith, Yuki Takahashi, Alongkone Phengsavanh, Tetsuyoshi Kariya, Yu Mon Saw, Nobuyuki Hamajima

**Affiliations:** 1 Department of Healthcare Administration, Nagoya University Graduate School of Medicine, Nagoya, Aichi, Japan; 2 Department of Healthcare and Rehabilitation, Ministry of Health, Vientiane Capital, Lao People’s Democratic Republic; 3 Department of Integrated Health Sciences, Nagoya University Graduate School of Medicine, Nagoya, Aichi, Japan; 4 Faculty of Medicine, University of Health Sciences, Vientiane Capital, Lao People’s Democratic Republic; Monash University, AUSTRALIA

## Abstract

Postpartum depression is a worldwide public health concern. The prevalence of postpartum depression is reported to be greater in developing countries than in developed countries. However, to the best of our knowledge, no papers on postpartum depression in the Lao People’s Democratic Republic have been published. In order to strengthen maternal and child health, the current situation of postpartum depression should be understood. This study aims to determine the prevalence of postpartum depression and identify factors associated with postpartum depression in Vientiane Capital, Lao People’s Democratic Republic. Study participants were 428 women 6–8 weeks postpartum who visited four central hospitals in Vientiane Capital for postnatal care from July to August 2019. Structured questionnaires were used to collect socio-demographic, obstetrical and infant, and psychiatric data about the women and their partners. The Edinburgh Postnatal Depression Scale (EPDS) was used to identify suspected cases of postpartum depression with the cut-off score of 9/10. Multivariable logistic regression was used to examine independent factors that were associated with suspected postpartum depression (EPDS ≥10). The mean age of the 428 women was 28.1 years, and the prevalence of suspected postpartum depression was 31.8%. Multivariable logistic regression using variables that were statistically significant on bivariate analyses indicated that three variables were associated with suspected postpartum depression: unintended pregnancy (AOR = 1.66, 95% CI 1.00–2.73, *P* = 0.049), low birth satisfaction (AOR = 1.85, 95% CI 1.00–3.43, *P* = 0.049), and depression during pregnancy (AOR = 3.99, 95% CI 2.35–6.77, *P* <0.001). In this study, unintended pregnancy, low birth satisfaction, and depression during pregnancy were independent risk factors for postpartum depression. These results suggest that the mental health of pregnant women should be monitored, and that health care services, especially family planning and supportive birth care, should be strengthened to prevent postpartum depression.

## Introduction

Postpartum depression (PPD) is defined as a non-psychotic episode of depression that may occur in women as early as four weeks after childbirth [1]. According to a meta-analysis and a systematic review of PPD, 13% of postnatal women globally experience mental disorders, and this proportion is noticeably higher, at 20%, in developing countries [2, 3]. PPD is considered a public health concern, as maternal mental disorders can affect the mother’s physical condition as well as the child’s growth, and family and social relationships. Poor maternal functioning due to mental suffering can affect a child’s later behavioral and cognitive development, and lead to poor physical growth and poor social engagement [4, 5]. Children of depressed mothers are more likely to be underweight with stunted growth, and experience more episodes of diarrhea and infectious illness [4]. A great deal of research has been conducted to identify risk factors that contribute to PPD, which may vary according to the social context and conditions. Many studies have reported that employment, level of education, first pregnancy, the child’s gender, a previous history of depression including during pregnancy, partner’s depression, dissatisfaction with the marital relationship, and lack of social support were the main factors associated with depression in women after delivery [6–8]. However, some studies have claimed that these factors were less important than socio-economic status and family income, which in turn are related to education and healthcare accessibility [8].

The Lao People’s Democratic Republic (Lao PDR), a landlocked country in Southeast Asia, is a low-middle-income country that experienced rapid economic growth in 2018 (per capita GDP of 2,542 USD) [9]. Numerous strategies and action plans have been implemented in order to strengthen maternal, newborn and child health [10], but none of them address to postpartum depression as information is lacking. Mental health statistics have been underreported due to the lack of specialists in the field of psychiatry and mental health services in the country [11]. There are only two hospitals (Mahosot Hospital and 103 Military Hospital) in Lao PDR that have psychiatry departments however these departments do not have psychiatric specialists. A mental health policy was introduced in 2018 to promote the mental health of the population by increasing the number of specialists and services [12].

To the best of our knowledge, there have been few studies on depression, but no studies on PPD in Lao PDR have been published. Therefore, the present study aims to determine PPD prevalence and identify factors associated with PPD in Vientiane Capital.

## Materials and methods

### Study design

This is a cross-sectional study that involved human participants (Lao women). The study was approved by the National Ethics Committee for Health Research in Lao PDR (No. 035/NECHR). Written informed consent was obtained from all women who were interviewed for this study. This study was conducted at four central hospitals (Mahosot Hospital, Mittaphab Hospital, Setthathirath Hospital, and Mother and Infant Hospital) in Vientiane Capital, Lao PDR because approximately 90% of the childbirths in Vientiane Capital were covered by the four hospitals [13]. Questionnaires were used to collect information from the study participants who were all women who visited the hospitals for postnatal care at 6–8 weeks postpartum from July to August 2019 and who agreed to participate in the study. Exclusion criteria were women who could not understand Lao language, had delivered twin babies, had thyroid disorders, and did not answer all the questions in the questionnaire. The mental health of mothers of twins can be affected by the double workload, and it has been reported that depression was associated with thyroid disorders [14]. Healthcare workers at the hospitals, such as physicians and midwives, were trained by Dr. Souphalak Inthaphatha (the first author) on methods of interviewing the participants and collecting data using the questionnaires without forcing the participants to answer on May 22–24, 2019.

A pilot study was conducted at Mittaphab Hospital involving 20 postpartum women in June 2019. It took approximately 15–20 minutes per interview and most questions were easily understood by the women. However, it was found that most Lao women did not visit doctors when they had mental problems. According to the results of the pilot study and comments from healthcare workers, the questionnaires were revised.

The predicted prevalence of PPD was assumed to be 20%, based on the prevalence of PPD in Thailand (16.8%) [15] and in other developing countries [2, 3]. Thailand is one of the neighboring countries to Lao PDR and Lao culture is similar to Thai culture. The minimum sample size was calculated to be 385 for the prevalence (20%) with the width of 95% confidence level less than 4%. After obtaining informed consent, a total of 433 women were interviewed in closed rooms in the presence of healthcare providers to ensure privacy. Five women were excluded because two mothers had twin babies, one had hyperthyroidism, one was a foreigner, and one had missing data. Finally, 428 postpartum women were included in this study.

### Questionnaire and measurement

The data were collected through structured questionnaires consisting of six sections: socio-demographic information (eight questions), obstetric and infant information (11 questions), information on the women’s partner and family including relationship satisfaction and availability of familial support (six questions), child-rearing information (four questions), history of depression of the study participants (six questions), and history of depression of their partners (two questions). Demographic data, health history, obstetric and infant information, and information on social support were obtained from both antenatal care logbooks and interviews.

Socio-demographic factors were categorized as follows. Ages of women were categorized into two groups (≤28 years and >28 years) based on the mean age. The ethnolinguistic groups that women belonged to were categorized into the following groups: Lao-Tai, Hmong-Mien, Mon-Khmer, and Chinese-Tibetan according to the ethnic categorization by the Lao government [16]. Marital status referred to women’s relationship with their partners. Women selected one from the following choices: single, married, cohabiting but not married, divorced, and widowed. Marital status was categorized into two groups (married/cohabiting and single/divorced). The education of women or their partners was based on the highest grade that they completed and was categorized into two groups (high school or lower and diploma/bachelor or higher). Area of residence was classified into urban or rural based on the district where the women lived. Household income was the monthly income of the women’s household, and it was classified into two groups (≤4,000,000 LAK and >4,000,000 LAK) based on the average household income.

The questions on obstetrics and infant factors inquired about the women’s last pregnancy and last live childbirth. Parity referred to the number of children women had. Answers on parity were grouped into two categories (one and two or more). Intended pregnancy was determined by asking the women whether they had planned the last pregnancy. Gestational weeks of the last childbirth were categorized into three groups (<37 weeks, 37–40 weeks, and >40 weeks) according to the criteria of preterm delivery and term delivery. Responses to birth satisfaction were divided into two groups: satisfied (very satisfied and satisfied) and not satisfied (neither satisfied nor dissatisfied, slightly dissatisfied, and very dissatisfied). Responses to relationships with their partner/mother/mother-in-law were divided into two groups: good (very good and good) and not good (neither good nor bad, bad, and very bad). “Passed away” was added to responses of relationship with their mother/mother-in-law.

### History of depression

According to the Diagnostic and Statistical Manual of Mental Disorders (DSM-5) [1], the diagnostic criteria of major depressive disorder is met when five or more of nine symptoms (depressed mood, markedly diminished interest or pleasure, significant weight loss, insomnia/hypersomnia, psychomotor agitation/retardation, fatigue, diminished ability to think, and recurrent thoughts of death) have been present during a 2-week period and at least one of the symptoms is either depressed mood or loss of interest or pleasure. There were no psychiatric specialists or general doctors who could diagnose depression based on DSM-5 in Lao PDR. Therefore, to identify depression, we developed two questions based on the criteria of major depression in DSM-5 to explore if the participants were (1) experiencing emotional stress and/or were unhappy, nearly every day for two weeks or longer, and (2) having recurrent thoughts of harming oneself or committing suicide. When the answer to either question was yes, the participant was considered as depressed. The two questions were used to identify the participant’s mental well-being before pregnancy, during pregnancy, and at the time of the interview; participant’s partner’s mental well-being before the interview was also considered.

### Edinburgh Postnatal Depression Scale

The Edinburgh Postnatal Depression Scale (EPDS) is a 10-item questionnaire used as a tool for screening for PPD by primary healthcare professionals [17]. The EPDS is used with different cut-off scores in different settings and cultures after validation in each country [15]. The EPDS in Lao language was found on a foreign website (https://www.mhcs.health.nsw.gov.au/publications/7005), but it was not used in this study because the process of translation was not described on the website. In this study, the EPDS was newly translated from English into Lao by two Lao translators (S1 Appendix), and then back-translated from Lao to English to ensure translation accuracy by two different Lao translators. This was followed by a pilot pre-test in 20 Lao postpartum women to determine if the questions could be understood by participants from different education backgrounds. The Lao EPDS was validated by the two questions that identified depression in women at the time of the interview but not by the psychiatric diagnosis because there were no psychiatric specialists in Lao PDR. Of the 428 women, 43 were considered as having depression at the time of the interview. The sensitivity and specificity at three cut-off scores of EPDS for depression in this study were 67.4% and 65.2% at 8/9, 60.5% and 71.4% at 9/10, and 55.8% and 80.8% at 10/11, respectively. The cut-off score of 9/10 was finalized to define “suspected PPD” (EPDS ≥10) in this study. The cut-off score of 9/10 is also recommended to detect minor and major depressive symptoms in primary healthcare settings among postpartum women in non-English speaking countries [18].

### Statistical analysis

For statistical analysis, all variables were divided into four main categories: 1) socio-demographic data on postpartum women, 2) obstetric and infant characteristics, 3) information on marital relationship and social support, and 4) depression history of women as well as their partners. Statistical Package for Social Sciences, version 25 (IBM SPSS Inc, Armonk, NY, USA) was used for data analyses. Chi-square test was performed to examine the relation between suspected PPD and each variable, but we used Fisher’s exact test when more than 20% of cells had expected frequencies <5. To identify independent risk factors for suspected PPD, multiple logistic regression analyses were performed using variables which showed statistically significant differences on the chi-square test or Fisher’s exact test. *P* value <0.05 was considered significant.

## Results

The mean age of the 428 postpartum women was 28.1 years (range, 16−44 years old). In terms of ethnicity, Lao-Tai was the main ethnolinguistic group (n = 403, 94.2%), and most women (n = 424, 99.1%) were married or living with a partner (Table 1). The mean household monthly income was 4,000,000 Lao Kip (LAK) (approximately 451 USD) ranging from 500,000 LAK (56.5 USD) to 70,000,000 LAK (7,903 USD). There was a considerable gap in household income as 291 women (68.0%) had a household income of 4,000,000 LAK or less. However, only 95 women (22.2%) reported that their household income was not enough to cover living costs. The proportion of women with only one child was 51.4% (n = 220), which was similar to the proportion of women with two or more children (Table 2). The last pregnancy had been planned in 305 women (71.3%) and only 14 women (3.3%) had complications during their last pregnancy. For most women childbirth had occurred at 37–40 gestational weeks (n = 295, 68.9%), they had delivered at health facilities (n = 423, 98.8%), and had vaginal delivery (n = 324, 75.7%). With regard to infant health, 50 infants (11.7%) had low birth weight (<2,500g), 18 infants (4.2%) had complications after delivery, and 22 infants (5.2%) had malnutrition with weight-for-age measurement below -2 standard deviations (SD) at six weeks old.

**Table 1:** Bivariate analysis of socio-demographic factors and suspected PPD among postpartum women.

Variables		Total	Suspected PPD	*P* value^c^
		N (%)	n (%)	
Age (years old)				0.466
	≤28	225 (52.6)	75 (33.3)	
	>28	203 (47.4)	61 (30.0)	
Ethnolinguistic group				0.073
	Lao-Tai	403 (94.2)	124 (30.8)	
	Others^a^	25 (5.8)	12 (48.0)	
Marital status				1.000^d^
	Married/cohabiting	424 (99.1)	135 (31.8)	
	Single/divorced	4 (0.9)	1 (25.0)	
Education				0.009
	High school or lower	212 (49.5)	80 (37.7)	
	Diploma/bachelor or higher	216 (50.5)	56 (25.9)	
Occupation				0.004
	Housewife	130 (30.4)	49 (37.7)	
	Government employee	97 (22.7)	22 (22.7)	
	Private company or NGO	80 (18.7)	17 (21.3)	
	Others^b^	121 (28.3)	48 (39.7)	
Area of residence				0.552
	Urban	223 (52.1)	68 (30.5)	
	Rural	205 (47.9)	68 (33.2)	
Household monthly income (LAK)				0.313
	≤4,000,000	291 (68.0)	97 (33.3)	
	>4,000,000	137 (32.0)	39 (28.5)	
Income adequacy for household living				0.651
	Enough	333 (77.8)	104 (31.2)	
	Not enough	95 (22.2)	32 (33.7)	

Abbreviations: PPD, postpartum depression; NGO, non-government organization; LAK, Lao Kip.

^a^Others are Hmong-Mien and Mon-Khmer.

^b^Others include businesswoman, merchant, labor worker and military.

^c^Chi-square test was used except in the variable where Fisher’s exact test was applied.

^d^Fisher’s exact test was used.

One USD = 8,947 LAK (April 1, 2020 rate)

**Table 2:** Bivariate analysis of obstetrics and infant factors and suspected PPD among postpartum women.

Variables		Total	Suspected PPD	*P* value^c^
		N (%)	n (%)	
Parity				0.040
	1	220 (51.4)	60 (27.3)	
	≥2	208 (48.6)	76 (36.5)	
Intended pregnancy				0.006
	Yes	305 (71.3)	85 (27.9)	
	No	123 (28.7)	51 (41.5)	
Complication during pregnancy				0.563^d^
	No	414 (96.7)	133 (32.1)	
	Yes	14 (3.3)	3 (21.4)	
Gestational weeks of delivery				0.215
	37–40 weeks	295 (68.9)	87 (29.5)	
	<37 weeks	35 (8.2)	15 (42.9)	
	>40 weeks	98 (22.9)	34 (34.7)	
Delivery place				0.037^d^
	Health facility	423 (98.8)	132 (31.2)	
	Others^a^	5 (1.2)	4 (80.0)	
Delivery method				0.088
	Vaginal delivery	324 (75.7)	110 (34.0)	
	Elective CS	41 (9.6)	7 (17.1)	
	Emergency CS	63 (14.7)	19 (30.2)	
Satisfaction with childbirth				0.002
	Satisfied	361 (84.3)	104 (28.8)	
	Not satisfied	67 (15.7)	32 (47.8)	
Infant gender				0.151
	Male	220 (51.4)	63 (28.6)	
	Female	208 (48.6)	73 (35.7)	
Infant birthweight				0.184
	<2,500g	50 (11.7)	20 (40.0)	
	≥2,500g	378 (88.3)	116 (30.7)	
Infant complication after delivery				0.710
	No	410 (95.8)	131 (32.0)	
	Yes	18 (4.2)	5 (27.8)	
Weight for age scale of infant at 6 weeks old^b^				0.072
	<-2SD	22 (5.2)	3 (13.6)	
	≥-2SD	399 (94.8)	127 (31.8)	

Abbreviations: PPD, postpartum depression; CS, caesarean section; SD, standard deviation.

^a^Others include home, car, and places other than health facilities.

^b^421 women answered because seven women did not have a record of baby’s weight or did not visit the hospital with their babies.

^c^Chi-square test was used except in the variables where Fisher’s exact test was applied.

^d^Fisher’s exact test was used.

The results of type of relationships and social support are shown in Table 3. Approximately half of the participants lived in nuclear families (n = 204, 47.7%). Of the 424 women who were married, 62.5% of their partners had a diploma or a bachelor’s degree, and the proportion of unemployed partners was only 1.4% (n = 6). Most women answered that they had a good relationship with their partners (n = 356, 84.0%), mothers (n = 382, 89.3%), and mothers-in-law (n = 330, 77.8%). In terms of social support, 330 women (77.1%) had someone who helped to take care of their babies other than their partners and 254 women (59.3%) had someone, other than family members, with whom they could discuss childrearing.

**Table 3:** Bivariate analysis of relationship and social support factors and suspected PPD among postpartum women.

Variables		Total	Suspected PPD	*P* value^d^
		N (%)	n (%)	
Family type				0.789
	Nuclear family	204 (47.7)	68 (33.3)	
	Staying with my parents	143 (33.4)	43 (30.1)	
	Staying with parents-in-law	81 (18.9)	25 (30.9)	
Partner’s education^a^				0.004
	High school or lower	159 (37.5)	64 (40.3)	
	Diploma/bachelor or higher	265 (62.5)	71 (26.8)	
Partner’s occupation^a^				0.042
	Unemployed	6 (1.4)	3 (50.0)	
	Government employee	140 (33.0)	38 (27.1)	
	Private company or NGO	118 (27.8)	31 (26.3)	
	Others^b^	160 (37.7)	63 (39.4)	
Relationship with partner^a^				<0.001
	Good	356 (84.0)	98 (27.5)	
	Not good	68 (16.0)	37 (54.4)	
Relationship with mother				0.032
	Good	382 (89.3)	115 (30.1)	
	Not good/passed away	46 (10.7)	21 (45.7)	
Relationship with mother-in-law^a^				0.002
	Good	330 (77.8)	93 (28.2)	
	Not good/passed away	94 (22.2)	42 (44.7)	
Family member who helps taking care of baby				0.204
	Partner	98 (22.9)	26 (26.5)	
	Other members	330 (77.1)	110 (33.3)	
Having someone to talk about childrearing besides family				0.319
	Yes	254 (59.3)	76 (29.9)	
	No	174 (40.7)	60 (34.5)	
Traditional hot/cold bed				0.054
	Yes	374 (87.4)	125 (33.4)	
	No	54 (12.6)	11 (20.4)	
Maternity leave^c^				0.067
	Yes	214 (71.8)	56 (26.2)	
	No	84 (28.2)	31 (36.9)	

Abbreviations: PPD, postpartum depression; NGO, non-government organization.

^a^424 women (not including four women who were single or divorced) answered.

^b^Others include businessman, merchant, labor worker, and military.

^c^298 women (other than housewives and women who were unemployed) answered.

^d^Chi-square test was used.

Most women used a traditional hot/cold bed after childbirth (n = 374, 87.4%). Using a hot/cold bed is a traditional practice among postpartum women. It gives opportunity for the women to rest, and stay away from daily chores for a certain period of time as well as to strengthen their social relations with their family and relatives [19]. Of 298 women who had jobs, 214 women (71.8%) took maternity leave.

Of the 428 women, 99 women (23.1%) had a history of depression before the last pregnancy (95% CI 0.19–0.27), 111 women (25.9%) had a history of depression during the last pregnancy (95% CI 0.22–0.30). Among 424 women who were married or cohabiting, 41 women (9.7%) had partners with a history of depression (95% CI 0.07–0.13) (Table 4).

**Table 4:** Bivariate analysis of history of depression and suspected PPD among postpartum women and their partners.

Variables		Total	Suspected PPD	*P* value^a^
		N (%)	n (%)	
Depression before pregnancy				0.001
	No	329 (76.9)	91 (27.7)	
	Yes	99 (23.1)	45 (45.5)	
Depression during pregnancy				<0.001
	No	317 (74.1)	74 (23.3)	
	Yes	111 (25.9)	62 (55.9)	
Partner’s depression^b^				0.002
	No	383 (90.3)	113 (29.5)	
	Yes	41 (9.7)	22 (53.7)	

Abbreviation: PPD, postpartum depression.

^a^Chi-square test was used.

^b^424 women (not including four women who were single or divorced) answered.

**Fig 1:**
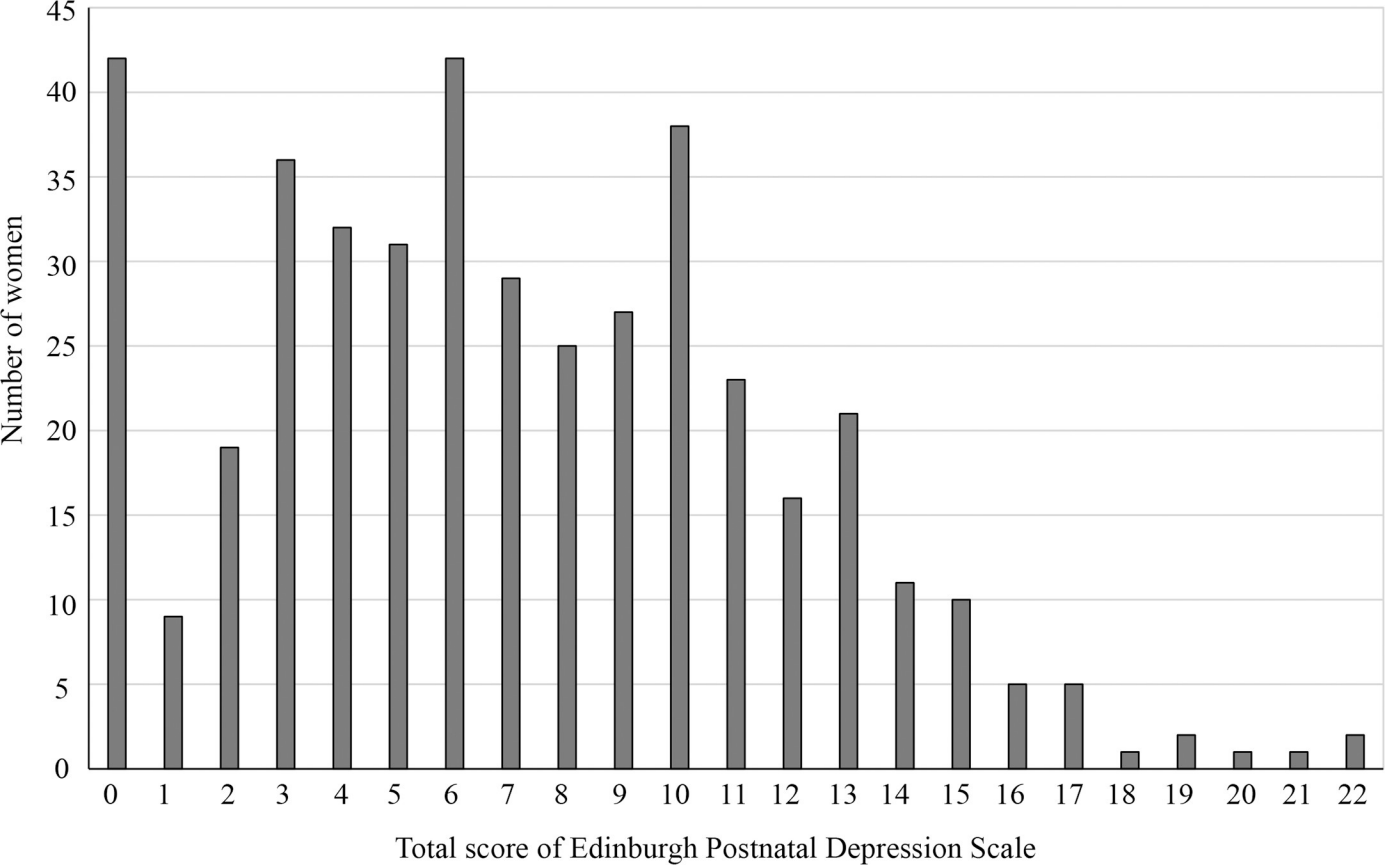
Distribution of the total score of Edinburgh Postnatal Depression Scale among postpartum women in Vientiane Capital.

The total EPDS score among participants ranged from 0 to 22 (Fig 1), and the mean was 7.1 (SD 4.6) with the median being 7.1. There were 136 women (31.8%) whose EPDS was 10 or higher, and they were defined as “suspected PPD” in this study. Comparing the characteristics of women with suspected PPD with those of the others, the factors significantly associated with suspected PPD were: higher education, occupation, parity of two or more, unintended last pregnancy, delivery place other than health facility, less satisfaction with childbirth, partner’s higher education, partner’s occupation, poor relationship with partner, poor relationship with mother, poor relationship with mother-in-law, depression before pregnancy, depression during pregnancy, and partner’s depression (Tables 1–4).

To identify independent risk factors for suspected PPD, we performed multivariate logistic regression analyses using the variables that were statistically significantly different on bivariate analyses (Table 5). Women whose last pregnancy was unintended (AOR = 1.66, 95% CI 1.00–2.73, *P* = 0.049), who were not satisfied with childbirth (AOR = 1.85, 95% CI 1.00–3.43, *P* = 0.049), and who had depression during pregnancy had significantly more suspected PPD than the others (AOR = 3.99, 95% CI 2.35–6.77, *P* <0.001). Women who had a poor relationship with their partners had a higher incidence of suspected PPD than the others but the difference was not significant (AOR = 1.88, 95% CI 0.99–3.56, *P* = 0.053).

**Table 5:** Multiple logistic regression on factors associated with suspected postpartum depression.

Variables		OR (95% CI)	*P* value	AOR^a^ (95% CI)	*P* value
Education					
	High school or lower	1 (reference)		1 (reference)	
	Diploma/bachelor or higher	0.58 (0.38–0.87)	0.009	0.91 (0.45–1.85)	0.800
Occupation					
	Housewife	1 (reference)		1 (reference)	
	Government employee	0.49 (0.27–0.88)	0.017	0.66 (0.30–1.47)	0.310
	Private company or NGO	0.45 (0.24–0.85)	0.014	0.68 (0.30–1.57)	0.368
	Others^b^	1.09 (0.65–1.81)	0.748	1.45 (0.79–2.64)	0.230
Parity					
	1	1 (reference)		1 (reference)	
	≥2	1.54 (1.02–2.31)	0.040	1.57 (0.97–2.51)	0.064
Intended pregnancy					
	Yes	1 (reference)		1 (reference)	
	No	1.83 (1.18–2.84)	0.007	1.66 (1.00–2.73)	0.049
Delivery place					
	Health facility	1 (reference)		1 (reference)	
	Others^c^	8.82 (0.98–79.66)	0.053	5.09 (0.48–53.51)	0.176
Satisfaction with childbirth					
	Satisfied	1 (reference)		1 (reference)	
	Not satisfied	2.26 (1.33–3.84)	0.003	1.85 (1.00–3.43)	0.049
Partner’s education					
	High school or lower	1 (reference)		1 (reference)	
	Diploma/bachelor or higher	0.54 (0.36–0.83)	0.004	0.95 (0.46–1.98)	0.898
Partner’s occupation					
	Unemployed	1 (reference)		1 (reference)	
	Government employee	0.37 (0.07–1.93)	0.239	0.89 (0.13–6.26)	0.902
	Private company or NGO	0.36 (0.07–1.86)	0.221	0.67 (0.10–4.66)	0.688
	Others^d^	0.65 (0.13–3.32)	0.604	0.89 (0.14–5.78)	0.906
Relationship with partner					
	Good	1 (reference)		1 (reference)	
	Not good	3.14 (1.85–5.34)	<0.001	1.88 (0.99–3.56)	0.053
Relationship with mother					
	Good	1 (reference)		1 (reference)	
	Not good/passed away	1.95 (1.05–3.63)	0.035	1.54 (0.74–3.21)	0.248
Relationship with mother-in-law					
	Good	1 (reference)		1 (reference)	
	Not good/passed away	2.06 (1.28–3.30)	0.003	1.06 (0.59–1.90)	0.855
Depression before pregnancy					
	No	1 (reference)		1 (reference)	
	Yes	2.18 (1.37–3.47)	0.001	1.31 (0.75–2.27)	0.347
Depression during pregnancy					
	No	1 (reference)		1 (reference)	
	Yes	4.16 (2.63–6.56)	<0.001	3.99 (2.35–6.77)	<0.001
Partner’s depression					
	No	1 (reference)		1 (reference)	
	Yes	2.77 (1.44–5.31)	0.002	2.06 (0.96–4.43)	0.064

Abbreviations: OR, odd ratio; AOR, adjusted odd ratio; CI, confidence interval; NGO, non-government organization.

Hosmer and Lemeshow = 4.301, -2 Log likelihood = 445,095, and R^2^ = 0.183.

^a^Adjusted for all variables listed in the table.

^b^Others include businesswoman, merchant, labor worker, and military.

^c^Others include home, car, and other places outside health facilities.

^d^Others include businessman, merchant, labor worker, and military.

## Discussion

To the best of our knowledge, this is the first report of a study on PPD in Lao PDR. The prevalence of suspected PPD among Lao women who visited central hospitals for postnatal care in Vientiane Capital was 31.8%, which was higher than the prevalence of PPD in other Asian countries. The mean prevalence of PPD in low- or middle-income countries is reported to be higher than that in high-income countries [3]. However, the reported prevalence in low- or middle-income countries varies depending on participants, study settings, and diagnostic methods; 7.8% in the provinces of Sri Lanka using EPDS ≥10, 8.4% in a nation-wide survey in Thailand using EPDS ≥13, 11.4% at maternity hospitals in Shanghai using EPDS ≥10, and 27.6% in Hanoi using EPDS ≥12 [7, 20–22]. These results suggest that women living in urban areas may have a higher prevalence of PPD. It has also been reported that the prevalence of postpartum mental disorders estimated by self-reported symptoms was significantly higher than that obtained by diagnostic assessments (20.8% vs. 16.1%) [3]. This study was conducted at central hospitals in the capital, and suspected PPD was defined based on the responses to the questions addressing self-reported symptoms [11]. The prevalence of PPD diagnosed by psychiatrists or among women in provinces in Lao PDR might be lower than 31.8%.

Factors associated with suspected PPD in multivariate analysis were unintended pregnancy, low satisfaction with childbirth, and depression during pregnancy. Previous studies reported that unintended pregnancy is associated with PPD [23, 24]. A prospective study in South Korea showed that unintended pregnancy contributed to PPD and parenting stress in mothers through marital conflict and low father’s participation in child care [25]. The impact of unintended pregnancy on PPD was highest at four months postpartum but the impact on parenting stress continued up to two years postpartum suggesting long-term influences on maternal and child health. In our study, 27.8% of women had unintended pregnancy and there was no difference between women who had only one child from the last pregnancy and women who had two or more children. Family planning is a cost-effective preventive method to reduce unintended pregnancies [26]. Public health facilities provide a family planning service free of charge in Lao PDR, but the policy should be strengthened to reduce unintended pregnancies.

A systematic review reported that negative birth satisfaction was significantly associated with PPD in most studies on birth experience [27]. The prospective cohort study suggested that post-traumatic stress disorder contributes toward PPD, which negatively influences satisfaction in a couple’s relationship [28]. Birth satisfaction is influenced by women’s expectations before childbirth, but is comprised of various factors, such as safety, support, respect, privacy, and involvement in decision making [29, 30]. A qualitative study reported that Lao women in rural areas prefer childbirth at home because of the support they receive from their family members and a preference for traditional childbirth practices [31]. In our study, five women, all of whom had childbirth at places other than health facilities, were satisfied with their childbirth, although they were more likely to develop PPD. Further studies on birth satisfaction using a global scale measurement of birth satisfaction are needed to understand the relationship between PPD and birth satisfaction in Lao women. Supportive care during childbirth can supersede pain and complicated birth [32]. The Lao government promotes institutional deliveries, therefore, healthcare workers, especially midwives, need to be trained to provide supportive care to women to enhance their confidence and comfort.

Several studies have reported that a mother’s mental well-being is associated with the relationship with their partners [6, 33, 34]. According to previous studies, couple relationship, birth satisfaction, unintended pregnancy, and PPD influence each other [25, 27, 28, 35]. Pregnancy or planning for pregnancy is the beginning of the transition to parenthood for couples, and this is not so only for first-time parents but also for second-time parents [36]. Couples need to adjust and be supportive of each other during the transition. However, couples are at risk of psychological problems and marital dissatisfaction because the transition causes stress in their lives and amplifies the differences within the couple [36–38]. In both mothers and fathers, negative interaction with partners influences depression during pregnancy as well as postpartum [35]. Providing programs on childbirth education and enhancing the co-parenting relationship of couples at health facilities may be effective in promoting parental mental well-being [39].

History of depression is a major risk factor for PPD [3, 6, 18, 40], and women who have experienced stressful or depressing life events at any time in their lives experience a significantly higher incidence of PPD [7, 20]. Some pregnant women might experience anxiety and depression from time to time and it might be difficult to diagnose especially during the first trimester [18]. If depression is not diagnosed or treated, it could last until the third trimester; depression during the late trimester is significantly associated with PPD [40]. The results in our study will be helpful for healthcare providers in Lao PDR to understand PPD and to identify women who may need mental care and support. Experienced healthcare workers, who were part of this study, were aware that there were some women who struggled with depression after childbirth but did not know how to screen for and treat PPD. It is important to improve training for primary healthcare providers to identify women at risk of PPD and those suffering from PPD as well as to increase awareness of PPD among pregnant and postpartum women and their families [41]. Further research on the experiences and the level of knowledge of PPD among primary healthcare providers will be needed to understand PPD in Lao PDR.

This study has some limitations. First, PPD was measured as suspected PPD using EPDS with the cut-off score being decided by questions specifically developed for this study. Both of the Lao version of EPDS and the two questions were not validated using diagnosis of depression by psychiatric specialists. Some women with suspected PPD in this study may not have been diagnosed with PPD if examined by psychiatrists. However, the questions were developed based on the criteria of major depression [1], and study participants were themselves aware of their suffering at the time of the interview. Second, the partner’s history of depression was determined by the study participants based on the observation of their partners. The responses might be affected by the relationship between the women and their partners. Third, the aim of this study was to identify factors associated with PPD but power calculation for associated factors was not performed in this study. Fourth, the results of this study may not be representative of all Lao women in Vientiane Capital, because we only included women who visited central hospitals for postnatal care. The women in this study had a higher socio-economic status and higher rates of skilled birth attendant and facility-based delivery compared to the women’s data in Vientiane Capital [42]. To understand the situation of PPD among Lao women, further studies need to be conducted including women in other communities and provinces, as well as validation of the EPDS by Lao psychiatrists.

In conclusion, the incidence of suspected PPD was 31.8% among 6–8-week postpartum women in Vientiane Capital, Lao PDR. Unintended pregnancy, low birth satisfaction, and depression during pregnancy were factors that were significantly associated with PPD. We believe that women should receive continuous support during pregnancy, including the postpartum period, to maintain their mental health. Health care service, especially family planning and supportive birth care, should be strengthened. Further research including a validated screening test for postpartum women in Lao PDR is warranted.

## Supporting information

S1 Appendix (PDF)

## References

[1] American Psychiatric Association. Diagnostic and statistical manual of mental disorders 5th ed Arlington VA: American Psychiatric Association; 2013.

[2] O'Hara MW, Swain AM. Rates and risk of postpartum depression—a meta-analysis. Int Rev Psychiatry. 1996;8(1):37–54. 10.3109/09540269609037816

[3] Fisher J, Cabral de Mello M, Patel V, Rahman A, Tran T, Holton S, et al Prevalence and determinants of common perinatal mental disorders in women in low- and lower-middle-income countries: a systematic review. Bull World Health Organ. 2012;90(2):139G–49G. Epub 2012/03/17. 10.2471/BLT.11.091850 22423165 PMC3302553

[4] Gelaye B, Rondon MB, Araya R, Williams MA. Epidemiology of maternal depression, risk factors, and child outcomes in low-income and middle-income countries. Lancet Psychiatry. 2016;3(10):973–82. Epub 2016/09/22. 10.1016/S2215-0366(16)30284-X 27650773 PMC5155709

[5] Feldman R, Granat A, Pariente C, Kanety H, Kuint J, Gilboa-Schechtman E. Maternal depression and anxiety across the postpartum year and infant social engagement, fear regulation, and stress reactivity. J Am Acad Child Adolesc Psychiatry. 2009;48(9):919–27. Epub 2009/07/25. 10.1097/CHI.0b013e3181b21651 . 19625979

[6] Lancaster CA, Gold KJ, Flynn HA, Yoo H, Marcus SM, Davis MM. Risk factors for depressive symptoms during pregnancy: a systematic review. Am J Obstet Gynecol. 2010;202(1):5–14. Epub 2010/01/26. 10.1016/j.ajog.2009.09.007 20096252 PMC2919747

[7] Do TKL, Nguyen TTH, Pham TTH. Postpartum Depression and Risk Factors among Vietnamese Women. Biomed Res Int. 2018;2018(4028913):4028913 Epub 2018/10/16. 10.1155/2018/4028913 30320133 PMC6167583

[8] Zaidi F, Nigam A, Anjum R, Agarwalla R. Postpartum Depression in Women: A Risk Factor Analysis. J Clin Diagn Res. 2017;11(8):QC13–QC6. Epub 2017/10/04. 10.7860/JCDR/2017/25480.10479 28969212 PMC5620853

[9] The World Bank. GDP capita (current US$)—Lao PDR 2019 [cited 2020 February 12, 2020]. Available from: https://data.worldbank.org/indicator/NY.GDP.PCAP.CD?end = 2018&locations = LA&start = 2012&view = chart.

[10] Ministry of Health of Lao PDR. National strategy and action plan for integrated services on reproductive, maternal, newborn and child health 2016–2025 Vientiane: Ministry of Health of Lao PDR; 2015.

[11] Ministry of Health of Lao PDR. Report on psychiatric service deliveries in 2018 Vientiane Capital: Ministry of Health of Lao PDR; 2018.

[12] Ministry of Health of Lao PDR. Mental health strategy by year 2020 Vientiane Capital: Ministry of Health of Lao PDR; 2012.

[13] Lao Statistics Bureau. Provisional report of the Fourth Lao Population and Housing Census 2015 Vientiane Capital: Ministry of Planning and Investment; 2015 Available from: https://laosis.lsb.gov.la/board/BoardList.do?bbs_bbsid = B404.

[14] Bergink V, Kushner SA, Pop V, Kuijpens H, Lambregtse-van den Berg MP, Drexhage RC, et al Prevalence of autoimmune thyroid dysfunction in postpartum psychosis. Br J Psychiatry. 2011;198(4):264–8. Epub 2011/02/24. 10.1192/bjp.bp.110.082990 . 21343331

[15] Limlomwongse N, Liabsuetrakul T. Cohort study of depressive moods in Thai women during late pregnancy and 6–8 weeks of postpartum using the Edinburgh Postnatal Depression Scale (EPDS). Arch Womens Ment Health. 2006;9(3):131–8. Epub 2005/12/06. 10.1007/s00737-005-0115-7 . 16329000

[16] Yokoyama S. The situation of ethnic minorities in Laos Lao Health Master Planning Study Progress Report 1. Vientiane Capital, Lao PDR: Ministry of Health and JICA; 2001 p. A6-1–A6-8.

[17] Cox JL, Holden JM, Sagovsky R. Detection of postnatal depression. Development of the 10-item Edinburgh Postnatal Depression Scale. Br J Psychiatry. 1987;150:782–6. Epub 1987/06/01. 10.1192/bjp.150.6.782 . 3651732

[18] Bennett HA, Einarson A, Taddio A, Koren G, Einarson TR. Prevalence of depression during pregnancy: systematic review. Obstet Gynecol. 2004;103(4):698–709. Epub 2004/03/31. 10.1097/01.AOG.0000116689.75396.5f . 15051562

[19] Barennes H, Simmala C, Odermatt P, Thaybouavone T, Vallee J, Martinez-Aussel B, et al Postpartum traditions and nutrition practices among urban Lao women and their infants in Vientiane, Lao PDR. Eur J Clin Nutr. 2009;63(3):323–31. Epub 2007/11/15. 10.1038/sj.ejcn.1602928 18000519 PMC3435433

[20] Panyayong B. Postpartum depression among Thai women: a national survey. J Med Assoc Thai. 2013;96(7):761–7. Epub 2013/12/11. PubMed . 24319843

[21] Fan Q, Long Q, De Silva V, Gunarathna N, Jayathilaka U, Dabrera T, et al Prevalence and risk factors for postpartum depression in Sri Lanka: A population-based study. Asian J Psychiatr. 2020;47(101855):101855 Epub 2019/11/17. 10.1016/j.ajp.2019.101855 . 31733601

[22] Gan Y, Xiong R, Song J, Xiong X, Yu F, Gao W, et al The effect of perceived social support during early pregnancy on depressive symptoms at 6 weeks postpartum: a prospective study. BMC Psychiatry. 2019;19(1):232 Epub 2019/07/31. 10.1186/s12888-019-2188-2 31357958 PMC6664519

[23] Upadhyay AK, Singh A, Singh A. Association between unintended births and risk of postpartum depression: Evidence from Ethiopia, India, Peru and Vietnam. SSM Popul Health. 2019;9:100495 Epub 2019/10/28. 10.1016/j.ssmph.2019.100495 31650000 PMC6804781

[24] Mercier RJ, Garrett J, Thorp J, Siega-Riz AM. Pregnancy intention and postpartum depression: secondary data analysis from a prospective cohort. BJOG. 2013;120(9):1116–22. Epub 2013/05/09. 10.1111/1471-0528.12255 23651010 PMC3708972

[25] Bahk J, Yun SC, Kim YM, Khang YH. Impact of unintended pregnancy on maternal mental health: a causal analysis using follow up data of the Panel Study on Korean Children (PSKC). BMC Pregnancy Childbirth. 2015;15:85 Epub 2015/04/17. 10.1186/s12884-015-0505-4 25881099 PMC4387588

[26] Cleland K, Peipert JF, Westhoff C, Spear S, Trussell J. Family planning as a cost-saving preventive health service. N Engl J Med. 2011;364(18):e37 Epub 2011/04/22. 10.1056/NEJMp1104373 . 21506736

[27] Bell AF, Andersson E. The birth experience and women's postnatal depression: A systematic review. Midwifery. 2016;39:112–23. Epub 2016/06/21. 10.1016/j.midw.2016.04.014 . 27321728

[28] Garthus-Niegel S, Horsch A, Handtke E, von Soest T, Ayers S, Weidner K, et al The Impact of Postpartum Posttraumatic Stress and Depression Symptoms on Couples' Relationship Satisfaction: A Population-Based Prospective Study. Front Psychol. 2018;9:1728 Epub 2018/10/05. 10.3389/fpsyg.2018.01728 30283380 PMC6157399

[29] Saisto T, Salmela-Aro K, Nurmi JE, Halmesmaki E. Psychosocial predictors of disappointment with delivery and puerperal depression. A longitudinal study. Acta Obstet Gynecol Scand. 2001;80(1):39–45. Epub 2001/02/13. 10.1034/j.1600-0412.2001.800108.x . 11167187

[30] Hildingsson I. Women's birth expectations, are they fulfilled? Findings from a longitudinal Swedish cohort study. Women Birth. 2015;28(2):e7–13. Epub 2015/02/24. 10.1016/j.wombi.2015.01.011 . 25700792

[31] Sychareun V, Hansana V, Somphet V, Xayavong S, Phengsavanh A, Popenoe R. Reasons rural Laotians choose home deliveries over delivery at health facilities: a qualitative study. BMC Pregnancy Childbirth. 2012;12:86 Epub 2012/08/29. 10.1186/1471-2393-12-86 22925107 PMC3449206

[32] Britton JR. The assessment of satisfaction with care in the perinatal period. J Psychosom Obstet Gynaecol. 2012;33(2):37–44. Epub 2012/05/05. 10.3109/0167482X.2012.658464 . 22554136

[33] Joshi D, Shrestha S, Shrestha N. Understanding the antepartum depressive symptoms and its risk factors among the pregnant women visiting public health facilities of Nepal. PLoS One. 2019;14(4):e0214992 Epub 2019/04/05. 10.1371/journal.pone.0214992 30947251 PMC6448918

[34] Chi X, Zhang P, Wu H, Wang J. Screening for Postpartum Depression and Associated Factors Among Women in China: A Cross-Sectional Study. Front Psychol. 2016;7:1668 Epub 2016/11/17. 10.3389/fpsyg.2016.01668 27847483 PMC5088192

[35] Figueiredo B, Canario C, Tendais I, Pinto TM, Kenny DA, Field T. Couples' relationship affects mothers' and fathers' anxiety and depression trajectories over the transition to parenthood. J Affect Disord. 2018;238:204–12. Epub 2018/06/11. 10.1016/j.jad.2018.05.064 . 29886200

[36] Figueiredo B, Conde A. Anxiety and depression symptoms in women and men from early pregnancy to 3-months postpartum: parity differences and effects. J Affect Disord. 2011;132(1–2):146–57. Epub 2011/03/23. 10.1016/j.jad.2011.02.007 . 21420178

[37] Le Strat Y, Dubertret C, Le Foll B. Prevalence and correlates of major depressive episode in pregnant and postpartum women in the United States. J Affect Disord. 2011;135(1–3):128–38. Epub 2011/08/02. 10.1016/j.jad.2011.07.004 . 21802737

[38] Lawrence E, Rothman AD, Cobb RJ, Rothman MT, Bradbury TN. Marital satisfaction across the transition to parenthood. J Fam Psychol. 2008;22(1):41–50. Epub 2008/02/13. 10.1037/0893-3200.22.1.41 18266531 PMC2367106

[39] Feinberg ME, Jones DE, Hostetler ML, Roettger ME, Paul IM, Ehrenthal DB. Couple-Focused Prevention at the Transition to Parenthood, a Randomized Trial: Effects on Coparenting, Parenting, Family Violence, and Parent and Child Adjustment. Prev Sci. 2016;17(6):751–64. Epub 2016/06/24. 10.1007/s11121-016-0674-z . 27334116 PMC10351206

[40] Ryan D, Milis L, Misri N. Depression during pregnancy. Can Fam Physician. 2005;51(8):1087–93. Epub 2005/08/27. PubMed 16121830 PMC1479513

[41] Trop J, Gendenjamts B, Bat-Erdene U, Doripurev D, Ganbold S, Bayalag M, et al Postpartum depression in Mongolia: A qualitative exploration of health care providers' perspectives. Midwifery. 2018;65:18–25. Epub 2018/07/22. 10.1016/j.midw.2018.06.020 . 30029083

[42] Lao Statistics Bureau. Lao Social Indicator Survey II 2017, Survey Findings Report Vientiane Capital: Lao Statistics Bureau and UNICEF; 2018.

