# Supplementary material for: Factors associated with postpartum depression among women in Vientiane Capital, Lao People’s Democratic Republic: A cross-sectional study
Source: PLoS One. 2020 Dec 4;15(12):e0243463. doi: 10.1371/journal.pone.0243463 (PMC7717544; doi:10.1371/journal.pone.0243463)
Supplement: S1 Appendix — (PDF) [file pone.0243463.s001.pdf]

S1 Appendix. Edinburgh Postpartum Depression Scale (in Lao language)

ກະລຸນາໝາຍອ້ອມວົງມົນໃສ່ໃນຂໍ້ທີ່ຄົນເຈັບຕອບວ່າຖືກກັບໂຕເອງທີ່ສຸດ ກ່ຽວກັບຄວາມຮູ້ສຶກຂອງເຂົາເຈົ້າໃນ**ຊ່ວງ 7 ມື້ ກ່ອນທີ່ຈະມາກວດຫຼັງເກີດນີ້**

1. ຂ້ອຍສາມາດຍິ້ມຫົວໄດ້ ແລະ ເຫັນສິ່ງຕ່າງໆເປັນເລື່ອງຕະຫຼົກດີ

☐ ຫຼາຍເທົ່າໆກັບທີ່ຂ້ອຍເຄີຍເປັນມາ

☐ ຂ້ອນຂ້າງໜ້ອຍກວ່າທີ່ເຄີຍເປັນມາ

☐ ດຽວນີ້ໜ້ອຍລົງຫຼາຍຢ່າງເຫັນໄດ້ແຈ້ງ

☐ ບໍ່ມີເລີຍ

2. ຂ້ອຍຕັ້ງຕາລໍຄອຍສິ່ງຕ່າງໆດ້ວຍຄວາມສຸກ(ຍິນດີ)

☐ ຫຼາຍເທົ່າໆກັບທີ່ຂ້ອຍເຄີຍເປັນມາ

☐ ຂ້ອນຂ້າງໜ້ອຍກວ່າທີ່ເຄີຍເປັນມາ

☐ ດຽວນີ້ໜ້ອຍລົງຫຼາຍຢ່າງເຫັນໄດ້ແຈ້ງ

☐ ບໍ່ມີຄວາມຍິນດີຫຍັງເລີຍ

3. ເມື່ອມີສິ່ງຜິດພາດເກີດຂຶ້ນ ຂ້ອຍມັກໂທດຕົວເອງໂດຍທີ່ບໍ່ຈຳເປັນ

☐ ບໍ່ເຄີຍເປັນຈັກເທື່ອ

☐ ບໍ່ຄ່ອຍເປັນປານໃດ

☐ ແມ່ນແລ້ວ ເປັນບາງຄັ້ງ

☐ ແມ່ນແລ້ວ, ສ່ວນໃຫຍ່ມັກໂທດຕົວເອງເກືອບຕະຫຼອດເວລາ

4. ຂ້ອຍມີຄວາມຮູ້ສຶກອຸກໃຈ ຫຼື ວິຕົກກັງວົນ ໂດຍບໍ່ມີເຫດຜົນ

☐ ບໍ່, ບໍ່ເຄີຍເປັນເລີຍ

☐ ບໍ່ຄ່ອຍເປັນ, ດົນໆເທື່ອໜຶ່ງ

☐ ແມ່ນແລ້ວ ມັກເປັນບາງຄັ້ງ

☐ ແມ່ນແລ້ວ, ມັກເປັນເລື້ອຍໆ

5. ຂ້ອຍມັກຢ້ານກົວ ຫຼື ຕົກໃຈກະວົນກະວາຍ ໂດຍບໍ່ມີເຫດຜົນ

☐ ບໍ່, ບໍ່ເຄີຍເປັນເລີຍ

☐ ບໍ່ຄ່ອຍເປັນປານໃດ

☐ ແມ່ນແລ້ວ, ເປັນບາງຄັ້ງ

☐ ແມ່ນແລ້ວ, ມັກເປັນຕະຫຼອດ

6. ມີບັນຫາສາລະພັດຢ່າງກຳລັງເກີດຂຶ້ນກັບຂ້ອຍຕອນນີ້

☐ ບໍ່ມີບັນຫາຫຍັງ, ຂ້ອຍສາມາດຮັບມືກັບບັນຫາໄດ້ດີຄືເກົ່າ

☐ ບໍ່ມີບັນຫາ, ສ່ວນໃຫຍ່ແລ້ວແມ່ນຂ້ອຍຈັດການກັບບັນຫາໄດ້ດີ

☐ ມີບັນຫາ, ບາງຄັ້ງກໍ່ຮັບມື ແລະ ຈັດການບໍ່ໄດ້ດີຄືແຕ່ເກົ່າ

☐ ມີບັນຫາ, ສ່ວນຫຼາຍແລ້ວແມ່ນຮັບບໍ່ໄຫວ, ຂ້ອຍບໍ່ສາມາດຈັດການກັບມັນໄດ້ເລີຍ

7. ຂ້ອຍບໍ່ມີຄວາມສຸກເລີຍ ຈົນເຮັດໃຫ້ຂ້ອຍນອນບໍ່ຫຼັບ

☐ ບໍ່ເຄີຍເປັນເລີຍ

☐ ບໍ່ຄ່ອຍເປັນປານໃດ

☐ ແມ່ນແລ້ວ, ເປັນບາງຄັ້ງ

☐ ແມ່ນແລ້ວ, ເປັນເກືອບຕະຫຼອດເວລາ

8. ຂ້ອຍຮູ້ສຶກໂສກເສົ້າ ແລະ ທຸກໃຈ

☐ ບໍ່ເຄີຍເປັນເລີຍ

☐ ບໍ່ຄ່ອຍເປັນປານໃດ

☐ ແມ່ນແລ້ວ, ເປັນບາງຄັ້ງ

☐ ແມ່ນແລ້ວ, ເປັນເກືອບຕະຫຼອດເວລາ

9. ຂ້ອຍຮູ້ສຶກບໍ່ມີຄວາມສູກຫຼາຍຈົນຮ້ອງໄຫ້

☐ ບໍ່ເຄີຍເປັນເລີຍ

☐ ບໍ່ຄ່ອຍເປັນປານໃດ

☐ ແມ່ນແລ້ວ, ເປັນບາງຄັ້ງ

☐ ແມ່ນແລ້ວ, ເປັນເກືອບຕະຫຼອດເວລາ

10. ຂ້ອຍເກີດມີຄວາມຄິດຢາກທຳຮ້າຍຕົນເອງ

☐ ບໍ່ເຄີຍຄິດເລີຍ

☐ ບໍ່ຄ່ອຍມີຄວາມຄິດນີ້ໃນຫົວເລີຍ

☐ ແມ່ນ, ມີຄວາມຄິດເປັນບາງຄັ້ງ

☐ ແມ່ນແລ້ວ, ມີຄວາມຄິດເກືອບຕະຫຼອດເວລາ

ການໝາຍຄະແນນ: ແມ່ນຈະໃຫ້ຄະແນນຕົວເລືອກຄຳຕອບຂອງແຕ່ລະຄຳຖາມເປັນ 0, 1, 2 ແລະ 3 ຕາມລຳດັບ. ໂດຍຄະແນນ EPDS ແມ່ນການເອົາຄະແນນທີ່ໄດ້ຈາກຄຳຕອບຂອງແມ່ຍິງໝາຍໃນແຕ່ລະຂໍ້ນັ້ນມາບວກໃສ່ກັນ ເປັນຄະແນນລວມທັງໝົດ.
